# Modified dindo-clavien system for registration of perioperative complications in children undergoing adenotonsillectomy

**DOI:** 10.3389/fped.2022.1049942

**Published:** 2022-12-30

**Authors:** Philipp Arens, Juliane Hardt, Julie Charlotte Angrick, Heidi Olze, Annekatrin Coordes

**Affiliations:** ^1^ Department of Otorhinolaryngology, Charité—Universitätsmedizin Berlin, Corporate Member of Freie Universität Berlin, Humboldt-Universität zu Berlin, and Berlin Institute of Health, Berlin, Germany; ^2^ Institute of Biometry and Clinical Epidemiology, Charité - Universitätsmedizin Berlin, Corporate member of Freie Universität Berlin, Humboldt-Universität zu Berlin, and Berlin Institute of Health, Berlin, Germany; ^3^ Institute for Integrative Medicine, Department for Human Medicine, Faculty of Health, Witten/Herdecke University, Herdecke, Germany

**Keywords:** complication reporting, pediatrics, adenotonsillectomy, dindo-clavien classification, sleep apnea

## Abstract

**Introduction:**

Surgical procedures in children are among the most commonly performed procedures in otolaryngology. Perioperative safety and documentation of complications are becoming increasingly important. This study investigates perioperative complications in a clinical cohort of children with adenotonsillar hyperplasia undergoing adenotonsillectomy using the standardized Dindo-Clavien reporting system.

**Patients and Methods:**

Retrospective evaluation of 402 children who underwent adenotonsillectomy between 2009 and 2015. Patient parameters including all perioperative complications were investigated.

**Results:**

In the study, 124 complications were found (106 mild, 16 severe). According to the Dindo-Clavien classification, 93 grade I, 15 grade II, 5 grade III, 11 grade IV and 0 grade V complications were documented. Complications were associated with additional diagnoses (*p* = 0.001), long-term medication intake (*p* = 0.003), duration of hospitalization (*p* < 0.001) and duration of surgery (*p* < 0.001), undergoing tonsillotomy (*p* = 0.022) or tonsillectomy (*p* < 0.001), differences in ASA score (*p* = 0.005) and differences in OSA-18 score (*p* = 0.011). Severe complications, classified as grade III and IV, were associated with premature birth (*p* = 0.026), additional diagnoses (*p* = 0.017), long-term medication intake (*p* < 0.001) and differences in ASA score (*p* =< 0.001).

**Conclusion:**

The Dindo-Clavien classification is a standardized reporting system which can also be used for surgical procedures in children with adenotonsillar hyperplasia. The system shows associations with clinical parameters and thus can help to identify subgroups at risk of severe complications.

## Introduction

Surgical procedures in children with adenotonsillar hyperplasia are among the most commonly performed procedures in otolaryngology, head and neck surgery (1). The most frequent indications are recurrent or chronic inflammation of the tonsillar tissue (with or without otitis media) and obstructive sleep-apnea (OSA) (2). The safety of these procedures is attracting increased attention. In children with OSA, there is often discussion about the need for an inpatient stay or admission to the intermediate care unit during the postoperative period (3–5). Furthermore, there is also a lack of standardized complication classifications in pediatric surgical patients, including children undergoing standardized surgery such as adenoidectomy (AT) and/or tonsillectomy/tonsillotomy (TE/TT).

Clavien et al. developed a classification system in 1992 for surgical complications in general surgery. Complications were classified into four grades depending on the therapy treating the complication (6). Dindo et al. modified the score and thereby focused on reporting permanently disabling and life-threatening complications (7).

Today, the modified Dindo-Clavien system is widely used to quantify patient outcomes in general surgery (8).

This study investigates perioperative complications in children with adenotonsillar hyperplasia undergoing AT and/or TE/TT using the standardized Dindo-Clavien reporting system.

## Methods

### Study design

The university hospital Charité—Universitätsmedizin Berlin is one of the largest children's clinic in Germany with children's intensive care units. Children with adenotonsillar hyperplasia who underwent AT and/or TE/TT at the Department of Otolaryngology at the Charité—Campus Virchow Klinikum between 2009 and 2015 were included in the study. The relevant data of all patients (aged 0–18) were extracted from the patients' files retrospectively. The study was conducted in accordance with the declarations of Helsinki and the guidelines of the Charité Berlin ethics committee (EA2/101/14).

All parents were routinely asked to complete the clotting questionnaire before surgery (9). If a coagulation disorder was suspected, patients were sent to the coagulation outpatient department.

All operations were performed under general anesthesia. Adenoidectomy was performed using the ring knife *via* a transoral approach under indirect mirror visualization. Tonsillectomies were performed using the cold dissection method and tonsillotomies with the monopolar needle. Bleeding lesions were gently coagulated with bipolar forceps. Patients received soft food postoperatively and pain medication as recommended. After adenoidectomy, the patients were monitored in hospital for one night and after tonsillotomy or tonsillectomy for three nights. Children with coagulation disorders were treated according to the recommendations of the coagulation department and were admitted for seven days. Antibiotic therapy was not prescribed prophylactically, except when local signs of inflammation or foetor ex ore were noticed.

### Measurements and variables

Collected data of the patients included: age, sex, height, weight, body mass index, additional diagnoses, long-term medication, length of hospital stay, duration of surgery, previous general surgeries, previous surgeries of the upper respiratory tract, premature birth complications, OSA-18 score and ASA score. To capture patient comorbidity as completely as possible, “additional diagnoses” were collected. Any acute or chronic condition independent of the surgery-defining diagnosis that was reported by the patient or patient's family at the admission visit and noted in the patient's record was included. We counted as “long-term medication” all medications that the patient took permanently and not for acute reasons. This information was extracted from the medication schedules that were kept as admission medication in the patient's file. Any deviation from the standard of care or standard treatment course during hospital stay was considered a “complication”. The entire inpatient stay period was evaluated.

Preoperatively, the parents filled in the ***OSA-18 Quality of Life Survey questionnaire*** (10). This questionnaire contains 18 quality of life items, which include sleep disorders, physical symptoms, mental complaints, daytime discomfort and patients' own fears. The OSA-18 total score including all 18 items ranges from 18 (no impact on quality of life) to 126 (major negative impact). A value >60 is considered abnormal in previous studies. The severity of the sleep-related breathing disorder recorded is divided into three categories: less likely (<60), possible (≥60–80) and high probability (>80) of the presence of OSA in a child. To evaluate a potential bias, patients with and without a completed OSA-18 questionnaire were compared.

The ***American Society of Anasthesiologists Physical Status Classification*** (ASA-PS) is an instrument for the preoperative determination of anesthetic risks. It records the physical condition of a patient and is divided into six categories. Group I describes healthy patients, group II is mild general disease, group III is severe general disease without performance limitations, group IV is severe, long-lasting general disease with long-lasting general disease with performance limitations, and Group V is a moribund patient who is expected to die within 24 h, with or without surgery. Group VI includes brain-dead patients (11).

Every complication was graded with the ***Dindo-Clavien-Score.*** The score classifies complications into five groups (see Table 1) (7). Every deviation from the standard treatment or the expected postoperative course was documented and regarded as a complication. Complications with grades III and IV were regarded as severe complications.

**Table 1:** Dindo-clavien classification of complications.

Dindo-Clavien classification
Grade I	Any deviation from the normal postoperative course without the need for pharmacological treatment or surgical, endoscopic and radiological interventions.
	Permitted therapeutic regimens include: drugs as antiemetics, antipyretics, analgetics, diuretics and electrolytes and physiotherapy. This grade also includes wound infections opened at the bedside.
Grade II	Pharmacological treatment required with other medications than the permitted complications of grade I; including blood transfusions and total parenteral nutrition.
Grade III	Requiring surgical, endoscopic or radiological intervention
IIIa	Intervention not under general anesthesia
IIIb	Intervention under general anesthesia
Grade IV	Life-threatening complication (including CNS complications)* requiring ICU-management
IVa	Single organ dysfunction (including dialysis)
IVb	Multiorgan dysfunction
Grade V	Death of a patient

ICU, intensive care unit; CNS, central nervous system.

### Statistics

In this exploratory study, the association of possible complicating or seriously complicating factors were investigated and reported according to SAMPL-Guidelines (12). Data were presented according to the STROBE checklist (see Supplementary Figure S1). All statistical analyses were done with IBM SPSS version 25 (Statistical Package for the Social Sciences). Continuous variables were summarized with medians and interquartile ranges (IQR) and examined with the Mann-Whitney U test (as none of them were normally distributed). Categorical variables were expressed as numbers and percentages and examined using the Chi-squared test or Fisher's exact test. Group differences were systematically tested with significance tests in awareness that this procedure is associated with an inflation of type I error. As this is an exploratory study, significance tests are only used for detecting potential differences. Hence, *p*-values described as significant for two-sided *p*-values ≤0.05 are only given as an orientation and not to be interpreted as confirmatory.

## Results

### Description of the patients

Between 2009 and 2015, 402 children with adenotonsillar hyperplasia underwent surgery in the Department of Otolaryngology. Data collection was complete for all investigated factors, the OSA-18 Questionnaire only was completed with 354 children (88.1%). The Supplementary Figure S2 shows the comparison of patients with vs. without completed questionnaires. 121 of 402 patients experienced one or more complications. In total, 124 complications were found. Three children had two independent complications (Tables 2,3). However, only the most severe complication was evaluated for statistical analysis (Table 4). 106 complications were wound- related and 15 were due to respiratory conditions. Thus, at least one complication occurred in 121 patients (30.1%). In 106 patients, the complication was mild (Dindo-Clavien grade I and II). In 15 patients a severe complication occurred (Dindo-Clavien grade III and IV). Grade V complications were not found.

**Table 2:** Number of total complications during hospital stay (3 patients had 2 independent complications).

Complications (Dindo-Clavien grade)	*n*
**Wound**	
• Wound infection or fever requiring antibiotic treatment and/or antipyretics (D-C I)	90
• Bleeding controlled with conservative intervention (D-C II)	11
• Bleeding controlled surgically (D-C III)	5
**Airway**	
• Perioperative respiratory problems requiring no specific therapy or ICU monitoring (D-C I)	1
• Perioperative respiratory problems requiring inhalative therapy (D-C II)	3
• Perioperative respiratory problems requiring re-intubation and/or ICU monitoring (D-C IV)	11
**Others**	
• Injury of the teeth (D-C I)	1
• Extravasate of the lower extremities (D-C I)	1
• Hypertensive derailment (D-C II)	1
**Total complications, *n***	**124**

D-C, Dindo-Clavien grade; *n*, number.

**Table 3:** Total complications according to the dindo-clavien classification.

Complications according to Dindo-Clavien	*n* = 124
Grade I	93
Grade II	15
Grade III	5
Grade IV	11
Grade V	0
Mild complications (D-C I and II)	108
Severe complications (D-C III and IV)	16

D-C, Dindo-Clavien grade; *n*, number.

**Table 4:** Patient characteristics and statistical analyses.

Characteristics	Total	No complication	With complications			Comparison 1: without vs. all complications		Comparison 2: severe complications vs. all other patients	
			Simple	Severe	All	test	*p*-value	test	*p*-value
	402 (100%)	281 (69.9%)	106	15	121 (30.1%)				
Age (months), median (IQR)	**55.0** **(37.8–83.0)**	56.0 (38.0–83.0)	53.5 (36.0–83.5)	36.0 (23.0–78.0)	52.0 (35.0–80.5)	U	0.521	U	0.133
Sex	** **								
Male, *n* (%)	**245** **(60.9)**	170 (60.5)	68 (64.2)	7 (46.7)	75 (62.0)	chi^2^	0.866	chi^2^	0.286
Female, *n* (%)	**157** **(39.1)**	111 (39.5)	38 (35.8)	8 (53.3)	46 (38.0)				
Body height (cm), median (IQR)	**107.0** **(97.0–124.0)**	108.0 (98.0–124.0)	105.5 (96.8–125.3)	97.0 (80.0–123.0)	105.0 (95.0–124.5)	U	0.632	U	0.132
Body weight (kg), median (IQR)	**18.0** **(14.4–26.0)**	18.0 (14.5–26.0)	18.0 (14.5–27.0)	16.0 (10.5–22.5)	17.5 (14.0–26.5)	U	0.796	U	0.110
Body mass index, median (IQR)	**16.2** **(14.7–18.5)**	16.3 (14.6–18.5)	16.1 (14.7–19.2)	16.1 (14.7–19.2)	16.1 (14.9–17.2)	U	0.651	U	0.766
Additional diagnoses *n* (%)	**119** **(29.6)**	69 (24.6)	41 (38.7)	9 (60.0)	50 (41.3)	chi^2^	**0** **.** **001**	Fisher	**0** **.** **017**
Long-term medication, *n* (%)	**51** **(12.7)**	26 (9.3)	17 (16.0)	8 (53.3)	25 (20.7)	chi^2^	**0** **.** **003**	Fisher	**<0** **.** **001**
Premature birth, *n* (%)	**32** **(8.0)**	19 (6.8)	9 (8.5)	4 (26.7)	13 (10.7)	chi^2^	0.249	Fisher	**0** **.** **026**
ASA score (score 1–6), median (IQR)	**1** **(1–2)**	1 (1–2)	1 (1–2)	2 (1–3)	1 (1–2)	U	**0** **.** **005**	U	<0.001
1 (%)	**281** **(69.9)**	208 (74.0)	68 (64.2)	5 (33.3)	73 (60.3)				
2 (%)	**99** **(24.6)**	61 (21.7)	34 (32.1)	4 (26.7)	38 (31.4)				
3 (%)	**20** **(5.0)**	11 (3.9)	4 (3.8)	5 (33.3)	9 (7.4)				
4 (%)	**2** **(0.5)**	1 (0.4)	0 (0.0)	1 (6.7)	1 (0.8)				
Surgical procedures:	** **								
Adenoidectomy, *n* (%)	**374** **(93.0)**	264 (94.0)	97 (91.5)	13 (86.7)	110 (90.9)	chi^2^	0.376	Fisher	0.281
Tonsillotomy, *n* (%)	**113** **(28.1)**	69 (24.6)	39 (36.8)	5 (33.3)	44 (36.4)	chi^2^	**0** **.** **022**	Fisher	0.770
Tonsillectomy, *n* (%)	**128** **(31.8)**	69 (24.6)	54 (50.9)	5 (33.3)	59 (48.8)	chi^2^	**<0** **.** **001**	Fisher	1,000
Paracentesis/tube, *n* (%)	**276** **(68.8)**	194 (69.0)	71 (67.6)	11 (73.3)	82 (68.3)	chi^2^	0.982	Fisher	0.785
Other surgeries, *n* (%)	**85** **(21.1)**	64 (22.8)	19 (17.9)	2 (14.3)	21 (17.5)	chi^2^	0.294	Fisher	0.743
Duration of surgery (min), median (IQR)	**23.0** **(16.0–33.0)**	20.0 (15.0–30.0)	27.0 (18.0–36.3)	24.0 (15.0–35.0)	27.0 (18.0–36.0)	U	**<0** **.** **001**	U	0.719
Duration of hospital stay (days), median (IQR)	**5** **(3–5)**	4 (3–5)	5 (5–6)	4 (3–6)	5 (5–6)	U	**<0** **.** **001**	U	0.542
Duration of extended stay (days), median (IQR)	**1.0** **(0.8–1.0)**	1.0 (0.0–1.0)	1.0 (1.0–2.0)	1.0 (0.0–2.0)	1.0 (1.0–2.0)	U	**<0** **.** **001**	U	0.298
Proportion with extended stay, *n* (%)	**302** **(75.1)**	202 (71.9)	89 (84.0)	11 (73.3)	100 (82.6)	chi^2^	**0** **.** **031**	Fisher	1.000
OSA-18 questionnaire score (18–126), median (IQR)	**56.5** **(41.0–72.0)**	54.0 (38.5–69.0)	59.0 (46.0–73.0)	57.0 (48.0–80.0)	58.0 (46.5–74.5)	U	**0** **.** **011**	U	0.443
OSA risk scale (0–2; 0 = unlikely, 1 = possible, 2 = very likely), median (IQR)	**0** **(0.0–1.0)**	0 (0.0–1.0)	0 (0.0–1.0)	0 (0.0–1.5)	0 (0.0–1.0)	U	0.113	U	0.607
OSA unlikely (%)	**208** **(58.8)**	148 (61.4)	53 (53.0)	7 (53.8)	60 (53.1)				
OSA possible (%)	**90** **(25.4)**	59 (24.5)	28 (28.0)	3 (20)	31 (27.4)	chi^2^	0.172^†^	chi^2^	0.937^†^
OSA very likely (%)	**56** **(15.8)**	34 (14.1)	19 (19.0)	3 (20)	22 (19.5)	chi^2^	0.257^‡^	Fisher	0.441^‡^

^†^ OSA possible and OSA very likely vs. OSA unlikely.

^‡^ OSA very likely vs. OSA unlikely and OSA possible.

IQR, interquartile range; *n*, number; OSA, obstructive sleep apnea; Fisher, Fisher's exact test; chi^2^, Chi-squared test; *U*, Mann-Whitney U test for independent groups.

The clinical data of the patients are summarized in Table 4. The median patient age was 55 months. 60.9% were male and 39.1% female. Adenoidectomies, tonsillotomies, tonsillectomies, and paracenteses were performed in 93.0%, 28.1%, 31.8% and 68.8% of patients respectively. 35.3% had undergone previous general operations and 19.7% previous operations of the upper airways. Other operations included surgery of the nasal conchae. The median inpatient duration (with IQR) was 5 (3–5) days, and the total range was 0–16 days. Nine children (2.2%) had coagulation disorders (e.g., von Willebrand factor syndrome, hemophilia) and were monitored regularly for 7 days after surgery. 75.1% of patients had an extended stay [median 1.0 (IQR 0.8–1.0) days]. The ASA score was determined as one and two in 69.9% and 24.6%.

### Which factors were associated with complications?

The presence of additional diagnoses (*p* = 0.001), long-term medication (*p* = 0.003), duration of surgery (*p* < 0.001), duration of hospital stay (*p* < 0.001), duration of extended stay (*p* < 0.001), differences in ASA score (*p* = 0.005), surgical procedures tonsillotomy (*p* = 0.022) and tonsillectomy (*p* < 0.001) and OSA-18 score (*p* = 0.011) were significantly associated with the occurrence of complications. 46.9% of the patients with complications had a possible or probable OSA compared with 38.6% without any complications. Age, sex, premature birth, height, weight, body mass index and previous operations were not associated with overall complications.

### What overall factors are associated with severe complications?

Severe complications were associated with premature birth (*p* = 0.026), and also the presence of additional diagnoses (*p* = 0.017), long-term medication (<0.001) and differences in ASA score (*p* < 0.001). Even serious complications were not associated with age, gender, height, weight, BMI and pre-operations.

## Discussion

This study investigated perioperative complications in children with adenotonsillar hyperplasia undergoing AT and/or TE/TT using the standardized Dindo-Clavien reporting system. In general surgery, the Dindo-Clavien classification system is widely used to quantify patient outcomes (8). In this study, the total complication rate per patient was 30.1% (121/402) and the rate of severe complications per patient was 3.7% (15/402). The total complication rate in this cohort was increased compared with the literature. The rate of severe complications (grade III and IV according to the Dindo-Clavien reporting system) was consistent with current literature (4, 13–18).

Various factors may have contributed to the high total complication rate. Firstly, the children in this cohort were less healthy compared to children with adenotonsillar hyperplasia undergoing AT in an ambulatory surgery center. 119 of 402 (29.6%) children had comorbidities, and a probable OSA did not count as independent comorbidity in our analysis (4, 14). Another aspect was the rigorous file review. Any deviation from the standard protocol was counted as a complication. Therefore, 74,4% (90/121) children were scored as Dindo-Clavien grade I for children with wound infection or fever requiring antibiotic treatment or antipyretics. In daily routines, these mild complications are sometimes not regarded as relevant by physicians (Table 2). However, one of the advantages of standardized complication classification (e.g., Dindo-Clavien Score) is a clear definition of complications.

The grading of complications may help to identify relevant patient subgroups. In this study, we were able to identify subgroups at increased risk of complications (e.g., comorbidities, long-term medication, children with a higher preoperative ASA-score and children with an increased OSA-18 questionnaire score). The relationship between comorbidities and complications is well known in pediatric literature. Amoils et al. found a significantly increased risk of respiratory complications in children with cardiovascular disease or respiratory disease who underwent tonsillectomy or adenoidectomy (19). The use of long-term medication indicates concomitant diseases; therefore, this cannot be assumed as an independent risk factor. Additionally, concomitant diseases influence the ASA score. OSA itself is a known risk factor for perioperative complications (3). In this context, our findings are interesting because even though the OSA-18 is not able to detect an OSA with diagnostic certainty (20), the questionnaire might be helpful to identify children at higher risk of perioperative complications when preoperative polysomnography is not available. The standardized grading system of the Dindo-Clavien Score enables identification of children with serious complication risks (e.g., premature birth). Premature birth has been described as a risk factor for complications in children undergoing ATE with a diagnosis of OSA (19). Thus, a standardized tool like the Dindo-Clavien Score can help to identify subgroups at risk of severe complications in this special cohort of patients.

According to the criteria for complication reporting in surgical patients, defined by Martin et al*.*, this study fulfilled nine out of ten criteria (21): definition of the data acquisition method, indication of the follow-up duration, definition of complications, registration of the mortality and morbidity rate, causes of death, indication of total complications and procedure-specific complications, severity grade used, length-of-stay data, and risk factors included in the analysis.

Limitations of this study from a methodological view are the size of the cohort and consequentially the low number of severe complications (grade III and IV).

Unfortunately, only 88.1% completed the OSA-18 questionnaire (see also Supplementary Figure S2). Children with completed questionnaires underwent tonsillectomy and tonsillotomy more frequently. These surgical procedures have a longer planned hospital stay and also the risk of complications is naturally higher. It can also be assumed that patients who undergo tonsillectomy as opposed to, for example, adenoidectomy alone are also more likely to suffer from OSAS. It can also be assumed that the parents of children with suspected OSAS are more willing to complete the OSA-18 questionnaire. Not fully explained is the fact that, contrary to expectations, the proportion of patients who unexpectedly stayed longer in the hospital is higher in the group of patients who did not complete the questionnaire. It is possible that risk underestimation took place beforehand.

A further limitation is the focus on the immediate postoperative period during the hospital stay. Outpatient postoperative course data were not included which may be helpful to evaluate the safety of surgical procedures as complications like bleeding occur later (22).

## Conclusion

The Dindo-Clavien classification is a standardized reporting system which can also be used for surgical procedures in children with adenotonsillar hyperplasia. Treatment with antibiotics or additional antipyretics in case of suspected wound infection or fever are deviations from the standard protocol and therefore count as mild complications (grade I according to Dindo-Clavien Score). This system shows associations with clinical parameters and thus can help to identify subgroups at risk of severe complications.

## Data Availability

The original contributions presented in the study are included in the article/**Supplementary Material**, further inquiries can be directed to the corresponding author/s.
