# Supplementary material for: Modified dindo-clavien system for registration of perioperative complications in children undergoing adenotonsillectomy
Source: Front Pediatr. 2022 Dec 30;10:1049942. doi: 10.3389/fped.2022.1049942 (PMC9837099; doi:10.3389/fped.2022.1049942)
Supplement: Supplementary file 1 [file Table1.docx]

**Supplemental figure 1:** STROBE Statement

|  | Item No | Recommendation | Page No |
| --- | --- | --- | --- |
| **Title and abstract** | 1 | (*a*) Indicate the study’s design with a commonly used term in the title or the abstract  *„Retrospective evaluation“ and “clinical cohort” is stated in the abstract.* | 1 |
|  |  | (*b*) Provide in the abstract an informative and balanced summary of what was done and what was found *Methods and results are stated.* | 1 |
| Introduction | | | |
| Background/ rationale | 2 | Explain the scientific background and rationale for the investigation being reported  *A rationale for the investigation is explained.* | 3 |
| Objectives | 3 | State specific objectives, including any prespecified hypotheses  *Objective is explained.* | 3 |
| Methods | | | |
| Study design | 4 | Present key elements of study design early in the paper  *Study design is explained*. | 3-4 |
| Setting | 5 | Describe the setting, locations, and relevant dates, including periods of recruitment, exposure, follow-up, and data collection  *Setting, location, dates, data collection and follow up is mentioned.* | 3-4 |
| Participants | 6 | (*a*) Give the eligibility criteria, and the sources and methods of selection of participants.  *Eligibility criteria/selection of participants and sources are mentioned.* | 4 |
|  |  | (*b*) For matched studies, give matching criteria and number of exposed and unexposed  *Not applicable.* | n. a. |
| Variables | 7 | Clearly define all outcomes, exposures, predictors, potential confounders, and effect modifiers. Give diagnostic criteria, if applicable *Variables that are not self-defining are explained in terms of definition and method of collection.* | 4-5 |
| Data sources/ measurement | 8* | For each variable of interest, give sources of data and details of methods of assessment (measurement). Describe comparability of assessment methods if there is more than one group *Data source and evaluation method are explained.* | 4-5 |
| Bias | 9 | Describe any efforts to address potential sources of bias  *Bias is addressed in the discussion section.* | 8 |
| Study size | 10 | Explain how the study size was arrived at *“All patients” of a defined time period were included.* | 3 |
| Quantitative variables | 11 | Explain how quantitative variables were handled in the analyses. If applicable, describe which groupings were chosen and why *Done* | 5 |
| Statistical methods | 12 | (*a*) Describe all statistical methods, including those used to control for confounding *Done* | 5 |
|  |  | (*b*) Describe any methods used to examine subgroups and interactions *Subgroup comparison presented in a comprehensible way (unifactorial comparisons), no multivariable models, therefore, no estimation and analysis of confounding and interactions.* | 5 |
|  |  | (*c*) Explain how missing data were addressed *Non-responder analysis of OAS-18 questionnaire* |  |
|  |  | (*d*) If applicable, explain how loss to follow-up was addressed *Limitation of the analysis to the hospital stay, as complete data was available. This is mentioned in the methods section.* | 4 |
|  |  | (*e*) Describe any sensitivity analyses  *None performed* | - |
| Results | | |  |
| Participants | 13* | (a) Report numbers of individuals at each stage of study—eg numbers potentially eligible, examined for eligibility, confirmed eligible, included in the study, completing follow-up, and analysed  *Described by specifying the inclusion period and complete inclusion of all patients in the period. Incomplete data are mentioned in the tables via the number of cases and addressed in the discussion.* | 6 |
|  |  | (b) Give reasons for non-participation at each stage  *Done in the discussion section* | 8 |
|  |  | (c) Consider use of a flow diagram *Not necessary because file analysis is retrospective without follow-up beyond the reporting period.* |  |
| Descriptive data | 14* | (a) Give characteristics of study participants (eg demographic, clinical, social) and information on exposures and potential confounders  *See Table 2-4* | Tab. 4 |
|  |  | (b) Indicate number of participants with missing data for each variable of interest *See Table 5. Missing data for OSA-18 questionnaire* | Tab. 5 |
|  |  | (c) Summarise follow-up time (e.g., average and total amount)  *Follow-up time = duration of stay. Mentioned in the methods section and in detail in Table 4* | Tab. 4 |
| Outcome data | 15* | Report numbers of outcome events or summary measures over time  *Presentation for the entire treatment period during hospital stay.* |  |

| Main results | 16 | (*a*) Give unadjusted estimates and, if applicable, confounder-adjusted estimates and their precision (e.g., 95% confidence interval). Make clear which confounders were adjusted for and why they were included  *Unadjusted estimates, no confounder adjustment due to basic nature of the analysis, no confidence intervals of the measures but significance tests for group differences* | 6,  Tab. 4 |
| --- | --- | --- | --- |
|  |  | (*b*) Report category boundaries when continuous variables were categorized  *Yes, done for OSA 18 questionnaire* | Table 4 |
|  |  | (*c*) If relevant, consider translating estimates of relative risk into absolute risk for a meaningful time period *No risk measures calculated. Measures of descriptive statistics were obtained.* |  |
| Other analyses | 17 | Report other analyses done—e.g. analyses of subgroups and interactions, and sensitivity analyses  *Subgroup analyses presented* | Page 6, Table 4 |
| Discussion | | | |
| Key results | 18 | Summarise key results with reference to study objectives  *Done at the first two paragraphs of the discussion section*. | 7 |
| Limitations | 19 | Discuss limitations of the study, taking into account sources of potential bias or imprecision. Discuss both direction and magnitude of any potential bias *Done* | 7 |
| Interpretation | 20 | Give a cautious overall interpretation of results considering objectives, limitations, multiplicity of analyses, results from similar studies, and other relevant evidence *Multiplicity of analyses addressed in method section (= type I error inflation) Other points covered the discussion section*. | 7-8 |
| Generalisability | 21 | Discuss the generalisability (external validity) of the study results  *Done* | 7-8 |
| Other information | | | |
| Funding | 22 | Give the source of funding and the role of the funders for the present study and, if applicable, for the original study on which the present article is based  *No funding*. |  |

*Give information separately for exposed and unexposed groups.

**Note:** An Explanation and Elaboration article discusses each checklist item and gives methodological background and published examples of transparent reporting. The STROBE checklist is best used in conjunction with this article (freely available on the Web sites of PLoS Medicine at http://www.plosmedicine.org/, Annals of Internal Medicine at http://www.annals.org/, and Epidemiology at http://www.epidem.com/). Information on the STROBE Initiative is available at http://www.strobe-statement.org.
