# Supplementary material for: Modified dindo-clavien system for registration of perioperative complications in children undergoing adenotonsillectomy
Source: Front Pediatr. 2022 Dec 30;10:1049942. doi: 10.3389/fped.2022.1049942 (PMC9837099; doi:10.3389/fped.2022.1049942)
Supplement: Supplementary file 2 [file Table2.docx]

**Supplemental figure 2:** Comparison between patients with and without a completed OSA-18 Questionnaire

| **variables** | **all patients** | **Patients … completed OSA-18 questionnaire (% with predictor)** | | **test** | **p-value** |
| --- | --- | --- | --- | --- | --- |
|  | **402 (100)** | **with (n=354)** | **without (n=48)** |  |  |
| age (months), median (IQR) | **55.0 (37.8-83.0)** | 55.5 (38.0-82.3) | 53.0 (33.3-86.8) | U | 0.938 |
| sex |  |  |  |  |  |
| male, n (%) | **245 (60.9)** | 221 (62.4) | 24 (50.0) | chi² | 0.134 |
| female, n (%) | **157 (39.1)** | 133 (37.6) | 24 (50.0) |  |  |
| additional diagnosis, n (%) | **119 (29.6)** | 101 (28.5) | 18 (37.5) | chi² | 0.267 |
| long-term medication, n (%) | **51 (12.7)** | 46 (13.0) | 5 (10.4) | chi² | 0.785 |
| body height (cm), median (IQR) | 107.0 (97.0-124.0) | 107.0 (97.0-125.0) | 106.0 (93.5-120.0) | U | 0.824 |
| body weight (kg), median (IQR) | 18.0 (14.4-26.0) | 18.0 (14.5-26.3) | 16.8 (13.2-24.8) | U | 0.426 |
| body mass index, median (IQR) | 16.2 (14.7-18.5) | 16.2 (14.7-18.5) | 15.6 (14.6-18.3) | U | 0.454 |
| previous surgeries, n (%) | **142 (35.3)** | 121 (34.2) | 21 (43.8) | chi² | 0.254 |
| previous surgeries of upper airways, n (%) | **79 (19.7)** | 69 (19.5) | 10 (20.8) | chi² | 0.979 |
| premature birth, n (%) | **32 (8.0)** | 27 (7.6) | 5 (10.4) | Fisher | 0.566 |
|  |  |  |  |  |  |
| ASA score (1 … 6 points), median (IQR) | 1 (1-2) | 1 (1-2) | 1 (1-2) | U | 0.179 |
| 1 (%) | **281 (69.9)** | 251 (70.9) | 30 (62.5) |  |  |
| 2 (%) | **99 (24.6)** | 86 (24.3) | 13 (27.1) |  |  |
| 3 (%) | **20 (5.0)** | 15 (4.2) | 5 (10.4) |  |  |
| 4 (%) | **2 (0.5)** | 2 (0.6) | 0 (0.0) |  |  |
|  |  |  |  |  |  |
| **Surgical procedures** |  |  |  |  |  |
| adenoidectomy, n (%) | **374 (93.0)** | 329 (92.9) | 45 (93.8) | Fisher | 1.000 |
| tonsillotomie, n (%) | **113 (28.1)** | 108 (30.5) | 5 (10.4) | chi² | **0.006** |
| tonsillectomy, n (%) | **128 (31.8)** | 124 (35.0) | 4 (8.3) | chi² | **<0.001** |
| paracentesis/tube, n (%) | **276 (68.8)** | 238 (67.4) | 38 (79.2) | chi² | 0.138 |
| other surgeries, n (%) | **85 (21.1)** | 74 (21.0) | 11 (22.9) | chi² | 0.903 |
|  |  |  |  |  |  |
| duration of the surgery (min), median (IQR) | 23.0 (16.0-33.0) | 16.0 (14.3-23.0) | 24.5 (15.0-40.0) | U | 0.903 |
| duration of hospital stay (days), median (IQR) | 5 (3-5) | 5 (3-5) | 3 (3-4) | U | **<0.001** |
| duration of extended stay (days), median (IQR) | 1.0 (0.8-1.0) | 1.0 (0.0-1.0) | 1.0 (1.0-1.0) | U | 0.109 |
| proportion with extended stay, n (%) | **302 (75.1)** | 259 (73.2) | 43 (89.6) | chi² | **0.022** |
|  |  |  |  |  |  |
| complications total, n (%) | **121 (30.1)** | 113 (31.9) | 8 (16.7) | chi² | **0.046** |
| severe complications, n (%) | **15 (3.7)** | 13 (3.7) | 2 (4.2) | Fisher | 0.697 |
|  |  |  |  |  |  |
| complications (Dindo-Clavien grade), median (IQR) | 0 (0.0-1.0) | 0 (0.0-1.0) | 0 (0.0-0.0) | U | **0.044** |
| 0, n (%) | **281 (69.9)** | 241 (68.1) | 40 (83.3) |  |  |
| 1, n (%) | **90 (22.4)** | 86 (24.3) | 5 (10.4) |  |  |
| 2, n (%) | **16 (4.0)** | 14 (4.0) | 1 (2.1) |  |  |
| 3, n (%) | **4 (1.0)** | 4 (1.1) | 0 (0.0) |  |  |
| 4, n (%) | **11 (2.7)** | 9 (2.5) | 2 (4.2) |  |  |

Abbreviations: U = Mann-Whitney U test; chi² = Chi-squared test; Fisher = Fisher’s exact test

This supplemental table includes patients who filled in the OSA-18 questionnaire compared with parents who did not. For most variables, both groups did not differ. Differing variables included patients undergoing tonsillectomy or tonsillotomy, duration of hospital stay, proportion of patients with extended stay, the number of total complications and the distribution of complications.
